# Investigation and Systematic Risk Assessment in a Typical Contaminated Site of Hazardous Waste Treatment and Disposal

**DOI:** 10.3389/fpubh.2021.764788

**Published:** 2021-10-27

**Authors:** Wenhui Zhu, Xintong Yang, Jun He, Xiahui Wang, Ran Lu, Zheng Zhang

**Affiliations:** Soil Environmental Protection Center, Research Center of Heavy Metal Pollution Prevention and Control, Chinese Academy for Environmental Planning, Beijing, China

**Keywords:** heavy metals, waste disposal site, simulative diffusion assessment, health risk assessment, contaminated site

## Abstract

A total of 214 sampling sites of a hazardous waste disposal center were surveyed in a two-stage pollution investigation, including soil boreholes and groundwater monitoring wells. Results showed that chemical oxygen demand (COD) (4.00–2930.00 mg/L), fluoride (0.07–9.08 mg/L), chromium (0.12–1.20 μg/L), nickel (0.15–459.00 μg/L), lead (0.10–10.20 μg/L), cadmium (Cd) (0.05–16.40 μg/L), and beryllium (0.06–3.48 μg/L) were detected in groundwater samples. For soils, Cd in soil (78.7 mg/kg) exceeded the risk screening value (65 mg/kg) for soil contamination of the second type of development land (GB36600-2018), and there remained the risk of leakage in the landfill detection investigation. Then, a health risk assessment was carried out. Based on the definitions of the groundwater exposure pathway (HJ 25.3-2019) and the pollution investigation of groundwater, the carcinogenic and non-carcinogenic risks of groundwater were generally considered to be negligible. The carcinogenic risk and non-carcinogenic risk of the concerned pollutant in soil for risk assessment (Cd) under the condition of reutilization exceeded the corresponding acceptable levels (1E-06 and 1). The (non-)carcinogenic risk of Cd mainly came from oral intake of soil and inhalation of soil particles under two conditions of reutilization and non-utilization, so on-site workers and surrounding residents should be properly protected from the mouth and nose to minimize the intake of pollutants from the soil and soil particles. The area of soil contaminated by Cd was about 630.58 m^2^, and the amount of pollution was about 1261.16 m^3^. The heavy metal pollution was only distributed in the depth range of 0–2 m, and the suggested risk control value of soil pollutants under the condition of reutilization for Cd was 56 mg/kg. Based on different pollution characteristics of soil, groundwater, and the landfill, targeted control measures were proposed.

## Introduction

A contaminated site, also known as a “brown field,” refers to a site that is contaminated by the production, management, treatment, and storage of toxic and harmful substances, the stacking or treatment and disposal of hazardous wastes, as well as mining activities, and is harmful to human health or the ecological environment. With the deterioration of terrestrial ecosystem and the reduction of land productivity, pollution in soil and groundwater becomes increasingly serious, which poses threats and challenges to the ecological environment, food safety, drinking water safety, regional ecological environment, human settlement environment health, sustainable economic and social development, and even social stability (1, 2), which need to be paid close attention to. The hazardous waste treatment and disposal center is a typical contaminated site. The main treatment and disposal methods of hazardous waste produced in the industrial production process include incineration and sanitary landfill, during which pollutants such as organic matter, fluoride, and heavy metals are produced (3–5). Chromium, nickel, lead, cadmium (Cd), and beryllium are known to cause various health effects, such as certain cancers, respiratory diseases, gastrointestinal disorders, and skin allergies (6–8). In the process of disposal, some pollutants can migrate *via* the atmosphere, water, and other media, so pollutants will enter the soil, accumulate in the soil, and diffuse in underground water (9–12), causing some adverse effects on the ecological environment and the health of residents around the disposal center. It is urgent to assess the environmental risks of contaminated sites in order to provide early warning for the development of these risks.

Risk assessment of contaminated sites is an important part of the framework system of site environmental management. On the one hand, it can guide the environmental investigation and monitoring of pollutants in contaminated sites and obtain key parameters of soil and groundwater. In addition, risk assessment can determine whether the risks are worthy of attention and calculate the remediation targets and pollution scope of specific sites. Pollutants migrate in soil, groundwater, and others in the contaminated site. Therefore, many scholars are studying the quality of soil and water environment around hazardous waste disposal sites and other contaminated sites. Surface pollutants can enter groundwater through leaching, leakage, runoff, and other ways, posing a threat to the nearby ecological environment and human health. The toxicity, bioaccumulation, and persistence of trace metal pollution in groundwater have been widely studied by researchers all over the world (13–16). In addition to affecting aquatic systems, trace metals also affect human health through consumption and skin contact with polluted water (7, 17). In recent years, more pieces of research on the characterization of space pollution level and potential health risk assessment come out, including pollution characteristics, key pollutants and regions, and current health risk level analysis (18–20). However, there are few studies on the adverse effects of groundwater migration in contaminated sites. In recent years, numerous research has been done in the field of soil heavy metal pollution and health risk assessment in contaminated sites at home and abroad (21–26). However, due to different technical and budgetary constraints, climate and environment, regional soil heterogeneity, receptor exposure characteristics, and other factors, there is no international agreement on the quantitative risk management framework of contaminated land (27–29). In particular, the contaminated sites of hazardous waste treatment and disposal may have a variety of contaminated sources, rich types of pollutants, and unknown degrees of toxicity and harmful effects, and there is a transmission of pollutants between the soil and water. Therefore, it is of great significance to explore the evaluation and management of these hazardous waste disposal centers in China, find out the deficiencies in the process of local technical guidance, and put forward corresponding suggestions.

The objectives of this study were: (1) to investigate the current characteristics (concentration) and affected areas of pollution in soil and groundwater in a typical contaminated site of hazardous waste treatment and disposal, identify characteristic pollutants, determine the causes and potential sources of pollution, and quantify the risk of landfill leakage; (2) evaluate the spatial carcinogenic and non-carcinogenic risks caused by each exposure pathways of characteristic pollutants under the condition of reutilization or non-utilization, and simulate the groundwater migration of pollutants of concern; (3) comprehensively analyze the spatial distribution, health risks, local background values and soil remediation cases at home and abroad of heavy metals in soil and groundwater, determine the areas and soil depth, earthwork volume and area that need remediation, and put forward comprehensive and feasible control values of polluted heavy metals in soil and targeted control measures based on different pollution characteristics of soil, groundwater and the landfill, so as to provide reference for pollution risk control of hazardous waste treatment and disposal sites.

## Materials and Methods

### Study Area and Pollution Identification

**Figure 1:**
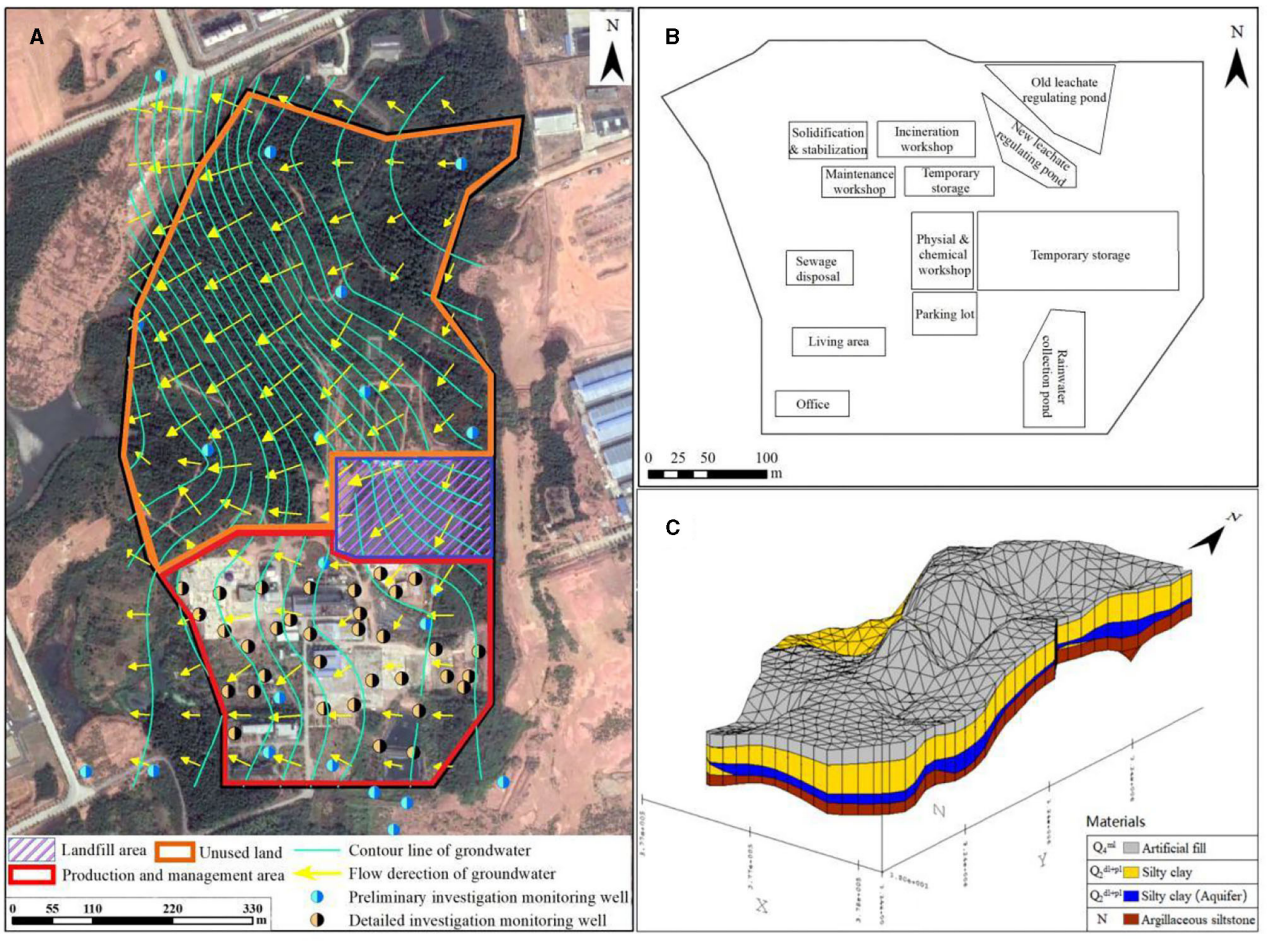
Flow direction of groundwater **(A)**, production and management area **(B)**, and strata classifications **(C)**.

The typical Hazardous Waste Disposal Center (HWDC) is located in a province in southern China. The HWDC is about 600 acres, of which the production and management area is about 175 acres, the unused land is about 380 acres, and the landfill area is about 45 acres, as shown in Supplementary Figure S1 in the support information S7. Geological and hydrogeological surveys (as shown in Figure 1) showed that the elevation of the HWDC was high in the north and low in the south. The stratigraphic structure was classified according to the sedimentary age and genetic type of the strata, and the top-down strata were divided into the artificial fill (${\text{Q}}_{4}^{\text{ml}}$), silty clay (${\text{Q}}_{2}^{\text{dl}+\text{pl}}$), and argillaceous siltstone (N). The groundwater type in the HWDC was pore phreatic water in loose rock, which mainly occurred in the ${\text{Q}}_{4}^{\text{ml}}$ pore. The groundwater flowed from the northeast to the southwest. The main lithology of this layer was silty clay, with weak permeability and continuous but irregular spatial distribution. Pore groundwater in this layer was poor, with poor mobility. According to the measured groundwater level elevation, the average hydraulic gradient of groundwater is about 0.018. According to the geotechnical laboratory test results, the range of permeability coefficient (recommended value) is 9.70E-06–5.22E-05 cm/s, and the range of groundwater seepage velocity (recommended value) is 1.51E-04–8.12E-04 m/day.

### Samples Collection and Detection

**Figure 2:**
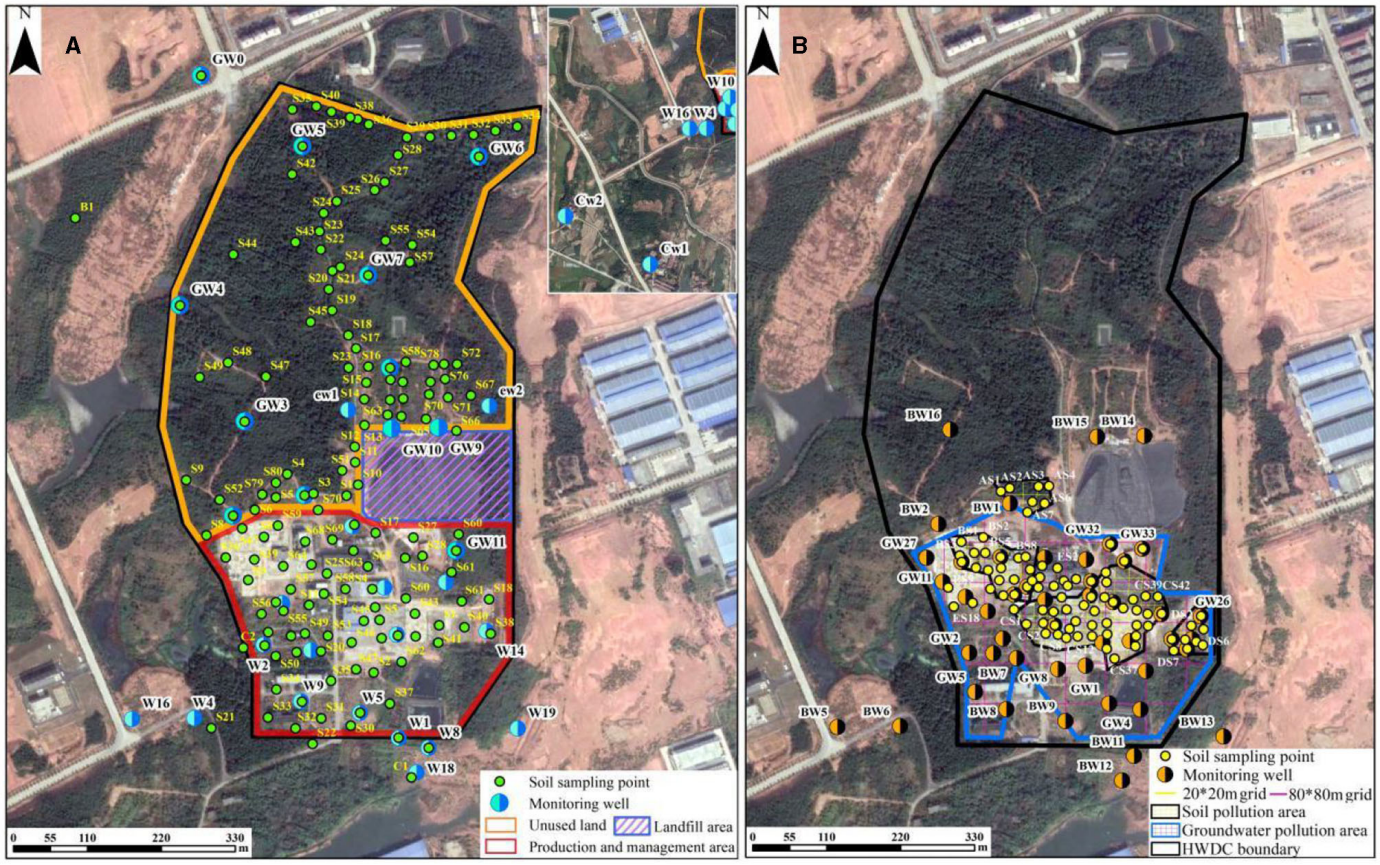
Sampling points distribution in the preliminary survey **(A)** and detailed investigation **(B)**.

In the preliminary investigation, based on the principle of “systematized layout and professional judgment,” sampling points were set in unused land and production and management area. Soil sampling holes were arranged according to 40 × 40 m grid density in the production and management area. A total of 73 soil sampling points (including one background point) were arranged, and 384 soil samples were collected. Twenty-four groundwater monitoring wells (including three original monitoring wells and two civil wells in the disposal center) were arranged according to the grid density of 80 × 80 m, and 27 groundwater samples (including three groundwater quality control samples) were collected in production and management area. For unused land, the preliminary investigation had a total of 81 soil sampling points. For groundwater sampling points, 12 groundwater monitoring wells (including one groundwater quality control sample) were arranged in the unused land according to the grid density of 150 × 150 m (the grid density of 40 × 40 m in the suspected pollution area in the unused land). A total of 266 samples were collected in unused land in the preliminary investigation, including 252 soil samples (including 28 quality control samples) and 14 groundwater samples (including two quality control samples). The distribution of sampling points in unused land and the production and management area is shown in Figure 2A.

According to the preliminary investigation results, the scope of the pollution survey had been significantly narrowed, and a further detailed investigation was carried out. A total of 141 sampling points were surveyed, including 90 soil boreholes and 51 groundwater monitoring wells. A total of 518 samples were collected in a detailed investigation, including 461 soil samples (including 45 quality control samples) and 57 groundwater samples (including 37 newly built groundwater monitoring wells, 14 original groundwater monitoring wells, and six quality control samples). Detailed investigation points are shown in Figure 2B.

In addition, the landfill was the key pollution source of the HWDC, and there remained a risk of pollution leakage after several years of discard. So, the corresponding site investigation was carried out in a detailed investigation. The landfill had a complete anti-seepage structure, so the traditional drilling survey was not suitable for the landfill. Therefore, the leakage detection equipment of the double electrode method was used to investigate the leakage risk of landfills. The double electrode method used two electrodes with an applied voltage to qualitatively determine whether there was leakage in the impervious layer of the landfill according to the circuit impedance. The field signal source of leakage detection with the double electrode method was 100–300 V, and the detection current was 100–500 mA. According to the 2–4 m point spacing, a total of 151 electrodes were arranged with five lines and a total length of 390 m.

### Health Risk Assessment

Two land-use plans, namely, the reutilization scenario and the non-utilization scenario, were considered. Then, hazard identification, exposure assessment, toxicity assessment, and risk characterization were carried out to determine whether the human health risk caused by soil and groundwater pollution exceeded the acceptable level. The risk control value of soil and groundwater pollution was calculated, and the quantity of contaminated soil to be remediated was estimated, which lays a solid foundation for the risk control and remediation scheme in the next step. *Technical guidelines for risk assessment of soil contamination of land for construction* (HJ 25.3-2019) stipulate 9 exposure pathways. The contaminants of concern identified by the preliminary and detailed investigation, heavy metals and fluoride, are not volatile. Therefore, the exposure pathways under the condition of reutilization were as follows: oral intake of soil, skin contact with soil, inhalation of indoor soil particles, and inhalation of outdoor soil particles. Correspondingly, the exposure pathways under the condition of non-utilization were oral intake of soil, skin contact with soil, and inhalation of outdoor soil particles.

First, the exposure dose of the identified contaminant is quantified. For carcinogenic pollutants, the exposure dose *via* the oral intake of soil for adults is calculated as Formula (1).

(1) $$\begin{array}{r} OISER_{ca}=\frac{OSIR_{a}\times ED_{a}\times EF_{a}\times ABS_{o}}{BW_{a}\times AT_{ca}}\times 10^{-6} \end{array}$$

where OISER_ca_ is the exposure dose *via* the oral intake of soil (carcinogenic effect), kg (soil)·kg^−1^ (body weight)·day^−1^; OSIR_a_ is the daily intake of soil for adults, mg·day^−1^; BW_a_ is the adult average weight, kg; EF_a_ is the adult exposure frequency, day·a^−1^; ED_a_ is the adult exposure cycle, a; ABS_o_ is the absorption efficiency factor of oral intake, dimensionless; AT_ca_ is the average time of carcinogenic effect, days. The recommended values of OSIR_a_, BW_a_, EF_a_, ED_a_, ABS_o_, and AT_ca_ are shown in Supplementary Table S1.

For non-carcinogenic pollutants, Formula (2) is used to calculate the exposure dose *via* the oral intake of soil for adults.

(2) $$\begin{array}{r} OISER_{nc}=\frac{OSIR_{a}\times ED_{a}\times EF_{a}\times ABS_{o}}{BW_{a}\times AT_{nc}}\times 10^{-6} \end{array}$$

where OISER_nc_ is the exposure dose *via* the oral intake of soil (non-carcinogenic effect), kg (soil)·kg^−1^ (body weight)·day^−1^; AT_nc_ is the average time of non-carcinogenic effect, days. The recommended value of AT_nc_ is shown in Supplementary Table S1. Formula (1) shows the meaning of OSIR_a_, BW_a_, EF_a_, ED_a_, ABS_o_. The exposure dose of (non-)carcinogenic pollutants *via* other pathways to be exposed to soil or groundwater can be found in the support information S1. Then, based on the toxicity parameters (as shown in the support information S2), the carcinogenic and non-carcinogenic risks were characterized. The carcinogenic risk of a single pollutant in soil *via* oral intake was calculated as Formula (3).

(3) $$\begin{array}{r} CR_{ois}=OISER_{ca}\times C_{sur}\times SF_{o} \end{array}$$

where CR_ois_ is carcinogenic risk *via* oral exposure to contaminated soil, dimensionless; C_sur_ is the concentration of pollutants in surface soil, mg·kg^−1^. C_sur_ values must be obtained according to site investigation. The meaning of OISER_ca_ and SF_o_ i is shown in Formula (1) and Formula (S7). The carcinogenic risks of a single pollutant *via* other exposure pathways for soil or groundwater are shown in support information S3. The hazard quotient of a single pollutant in contaminated soil *via* oral intake was calculated as Formula (4).

(4) $$\begin{array}{r} HQ_{ois}=\frac{OISER_{nc}\times C_{sur}}{RfD_{o}\times SAF} \end{array}$$

where HQ_ois_ is the hazard quotient *via* oral exposure to contaminated soil, dimensionless; SAF is the distribution coefficient of reference dose when exposed to soil, dimensionless. The meaning of OISER_nc_, C_sur_, and RfD_o_ is shown in Formula (3) and Formula (S8). The hazard quotients of a single pollutant *via* other exposure pathways for soil or groundwater are shown in support information S4. Based on the carcinogenic risk (total carcinogenic risk) or hazard quotient (hazard index) of various pollutants *via* different exposure pathways to soil or groundwater above, the carcinogenic risk and hazard index of all pollutants are calculated. The carcinogenic risk of all pollutants of concern *via* all exposure pathways is calculated as Formula (5).

(5) $$\begin{array}{r} CR_{sum}=\overset{n}{\underset{i=1}{\sum }}CR_{i} \end{array}$$

where CR_sum_ is the total carcinogenic risk of all pollutants (the number of kind is n) of concern, dimensionless. The definition of CR_i_ is shown in Formula (S11). The hazard index of all pollutants of concern *via* all exposure pathways is calculated as Formula (6).

(6) $$\begin{array}{r} HQ_{sum}=\overset{n}{\underset{i=1}{\sum }}HI_{i} \end{array}$$

where HQ_sum_ is the hazard index of all pollutants (the number of kind is n) of concern, dimensionless. The definition of HI_i_ is shown in Formula (S14).

### Simulation and Prediction Model of Pollutants in Groundwater

Groundwater modeling system (GMS 10.4) is a visual three-dimensional groundwater simulation software package. Modflow is a three-dimensional finite-difference groundwater flow model. MT3DMS is the most widely used three-dimensional groundwater solute transport simulation model. In the GMS software package, MT3DMS can be seamlessly connected with Modflow, supporting all hydrological and discrete characteristics of Modflow, which is the most widely used numerical model of solute transport at home and abroad. According to the pollution distribution of each pollutant and groundwater flow model, aquifer parameters, initial conditions, and boundary conditions are substituted into the water quality model; permeability coefficient is 0.05 m/d, the rainfall recharge rate is 3.22E-04 m/day, effective porosity is 3.00E-01, boundary discharge is 1.299 m^3^/day, dispersion is 4.4 m, and heavy metal soil-water allocation coefficients (Kd) are 1.50E+02 L/kg for fluoride, 1.50E+01 L/kg for Cd, and 1.60E+01 L/kg for a nickel. Modflow and MT3DMS models are used to jointly run the water flow and water quality model, and the prediction results of pollutant migration and transmission are obtained.

### Multivariate and Geostatistical Methods

SPSS 16.0 software (IBM, Armonk, NY, USA) was used for logging data and calculation. In order to analyze the characteristics of heavy metals, the basic statistical parameters such as average value, extreme value, detection rate, exceeding the rate, and exceeding time were calculated. Using a geographic information system (GIS) (ArcGIS 9.3 software), the concentration of toxic metals at each sampling point of groundwater in the disposal center on the plane map and the spatial distribution characteristics of toxic metals pollution in the soil of the whole region were demonstrated. IDW (inverse distance weighted) uses a specific number of nearest points and is then weighted according to their distance to the interpolated point (30–32). IDW method was used to draw the spatial distribution map of the toxic metals in the soil of the disposal center, so as to clearly show the spatial variation and spatial pattern of heavy metal concentrations in the study area.

## Results and Discussion

### Pollutant Concentrations in the Groundwater and Soil

#### Groundwater

Detailed investigation showed that chemical oxygen demand (COD) (4.00–2930.00 mg/L), fluoride (0.07–9.08 mg/L), chromium (0.12–1.20 μg/L), nickel (0.15–459.00 μg/L), lead (0.10–10.20 μg/L), Cd (0.05–16.40 μg/L), and beryllium (0.06–3.48 μg/L) were detected in groundwater samples, and the detection rate ranged from 6 to 100%. Among them, COD, fluoride, nickel, and Cd exceeded the corresponding Class IV water quality standards in *Standard for groundwater quality* (GB/T 14848-2017) (10 mg/L for COD, 2 mg/L for fluoride, 100 μg/L for nickel, and 10 μg/L for Cd). The pH value of the GW14 sample was 5.47, slightly lower than the standard value of 5.5. The groundwater samples exceeded the COD standard (GB/T 14848-2017) (10 mg/L) by 81%, and the maximum detectable concentration was 2,930 mg/L, which exceeded 10 mg/L by 292 times. The exceeding rates of fluoride and nickel samples were 8%, and the maximum exceeding times were 3.54 times and 3.59 times, respectively. Cd only has a single exceeding point, GW29 (16.40 μg/L), and the exceeding time was 0.64 times.

**Figure 3:**
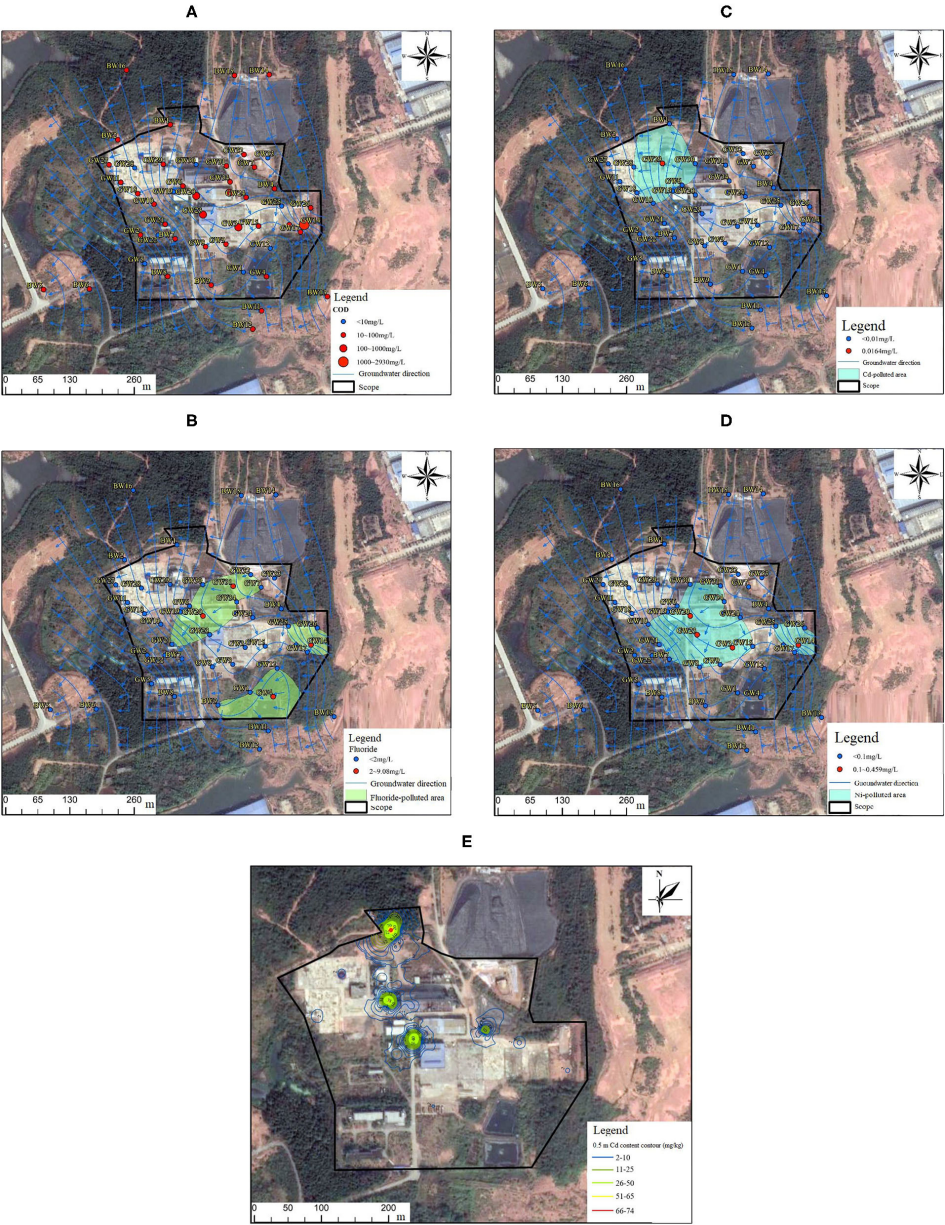
Spatial characterization of the sampling points exceeding COD **(A)**, fluoride **(B)**, Cd **(C)**, and nickel **(D)** standard in groundwater (GB/T 14848-2017) and spatial distribution of Cd pollution in 0.5 m soil (GB36600-2018) **(E)**.

The sampling points exceeding the Class IV water quality standards of COD (GB/T 14848-2017) and spatial distribution of groundwater COD are shown in Figure 3A. Figure 3A shows that the COD of the upper, middle, and lower reaches of the groundwater in the disposal center generally exceeded the standard. COD increased significantly in the middle reaches of the production management area, especially high in the original physical and chemical workshop (GW23), the north side of the physical and chemical workshop (GW20 and GW24) and the east side of the original temporary storage area (GW3 and GW14), and the exceeding ratio was more than 100 times. The COD concentration of BW5 and BW6 monitoring wells downstream slightly exceeded the standard, <10 times. The historical hazardous waste management activities of the disposal center may contribute to the excessive COD in groundwater. There was also a certain excessive COD upstream of groundwater. The disposal center is located in the gathering area of three leading industries which are textile and clothing, electronic information, and new energy and new materials. The exceeding standard of COD may also be a regional problem in the gathering area. However, the significant high concentration of COD in the production and management area also indicated the possibility of organic pollution in the groundwater of the HWDC, including volatile organic compounds (VOCs), semi-volatile organic compounds (SVOCs), and total petroleum hydrocarbons (TPH), which is worthy of attention in the future risk control strategies.

The sampling points exceeding Class IV water quality standards of fluoride in *Standard for groundwater quality* (GB/T 14848-2017) and spatial distribution of groundwater fluoride are shown in Figure 3B. Figure 3B shows that fluoride mainly slightly exceeded the standard, which was mainly distributed in the incineration workshop (GW31), the original temporary storage in the north of the physical and chemical workshop (GW20), the temporary storage in the east of the physical and chemical workshop (GW14), and the local area around the initial rainwater collection pool (GW4). The sampling points exceeding Cd standard (GB/T 14848-2017) and spatial distribution of groundwater Cd are shown in Figure 3C. Figure 3C shows that Cd pollution was mainly distributed in the surrounding area of the original solidification/stabilization workshop (GW29), exceeding Cd standard slightly. The sampling points exceeding nickel standard (GB/T 14848-2017) and spatial distribution of groundwater Cd are shown in Figure 3D. Figure 3D shows that the nickel-contaminated area was mainly concentrated in the northern side of the original temporary storage (GW20), the physical and chemical workshop (GW23), and the eastern side of the physical and chemical workshop temporary storage (GW3 and GW14). In the sewage treatment system of the disposal center, the unqualified discharge of production wastewater and domestic wastewater may lead to heavy metal pollution in water ponds. In addition, hazardous landfills may have a history of leakage, which may cause heavy metal pollution to groundwater downstream.

#### Soil

The preliminary investigation found that in the production and management area, for the soil, the total chromium of the site S10 located in the stabilization/solidification workshop area exceeded the standard. The total chromium evaluation standard refers to the Dutch soil and groundwater intervention value standard which was 380 mg/kg. But the maximum concentration of total chromium in this study was 1,170 mg/kg, far below the soil screening value (2,500 mg/kg) published in China. Thus, it was appropriate to consider the total chromium below the standard. In the unutilized area, Cd only in the surface S3 (0.5 m) (78.7 mg/kg) exceeded the risk screening value (65 mg/kg) for soil contamination of the second type of development land in *Soil environmental quality-Risk control standard for soil contamination of development land* (GB36600-2018) 0.21 times, and it was below the intervention value (172 mg/kg). According to the statistical analysis of the test results of soil samples, the excessive Cd samples only exist at 0.5 m sampling depth in the surface soil layer. According to the sampling depth of soil samples, the IDW method was used in ArcGIS to hierarchically characterize the spatial distribution of exceeding Cd pollution in soil, shown in Figure 3E. It can be seen from Figure 3E that Cd pollution in the 0.5 m soil layer was only distributed at the junction of the production management area and unused land on the north side. The pollution may come from road transport litter or dust. Incineration workshops were used to incinerate waste, and atmospheric emissions may lead to heavy metal pollution in the surrounding soil. For the production and management area, production workshops (incineration, material/chemical, and stabilization/solidification workshops) and temporary storage are directly exposed to hazardous waste, whereas facilities and equipment, site walls, and ground can be clung to a small amount of residual hazardous waste. In addition, varying degrees of damage can happen to buildings, and rainwater helps the leaching and infiltration of hazardous waste, causing heavy metal pollution of soil.

Detailed investigation showed that fluoride (331–2,060 mg/kg), chromium (24–394 mg/kg), nickel (7–546 mg/kg), lead (3.3–204 mg/kg), Cd (0.01–51.3 mg/kg), and beryllium (0.82–6.83 mg/kg) were detected in soil samples, and the detection rate ranged from 97.84 to 100%. The detection rate of Cd was 97.84%, and the detection rates of nickel, lead, Cd, and beryllium were all 100%. Chromium, nickel, lead, Cd, and beryllium contents were all below their corresponding standards.

### Leakage Risk of Landfill

According to the landfill leakage detection method and parameters in section Samples Collection and Detection, the impedance test distribution of landfill leakage detection was calculated, as shown in Supplementary Figure S2 in support information S8. Test results in Supplementary Figure S2 showed that the field signal source was 100–300 V, the detection current was 100–500 mA, and the impedance between the two electrodes of the landfill impervious layer was 1.1 k−1.2 k. There was a good electrical conductivity between the impervious layers, and the high-density polyethylene (HDPE) film of the impervious layer might have been damaged. In addition, Supplementary Figure S2 also showed that the overall conductivity of the landfill area on the east side was stronger than that on the west side, and the leakage risk of the impervious layer on the east side was higher than that on the west side. Groundwater monitoring data in section Groundwater showed that no significant pollution leakage was observed in the landfill, which may be due to the implementation of the surface coverage with HDPE membrane in this landfill, blocking the downward migration of pollutants resulting from the infiltration of large amounts of rainwater.

### Multi-Scenario Health Risk Assessment of Heavy Metals

#### Groundwater

Fluoride was the concern pollutant in groundwater that needs risk assessment, and the exceeding points were GW4, GW14, GW20, and GW31. The other two contaminants of concern were nickel, with excessive points GW3, GW14, GW20, and GW23 and Cd with GW29. The above pollutants are not volatile, therefore, according to the *Technical guidelines for risk assessment of soil contamination of land for construction* (HJ 25.3-2019), there was no corresponding exposure pathway, and the probability of human health risk caused by pollutants in groundwater *via* drinking groundwater was rather small. Therefore, it was considered that the carcinogenic risk and non-carcinogenic risk under the condition of reutilization or non-utilization were 0, so it was not necessary to consider the risk control value. But the risk of groundwater migration and diffusion must be paid attention to.

#### Soils

The concern pollutant for risk assessment was Cd, and the corresponding sampling point was S3.

1. Risk characterization results under the condition of reutilization
  The health risks of the pollutants in the soil under the condition of reutilization are shown in Tables 1, 2. It can be seen from Tables 1, 2 that under the condition of reutilization, the total carcinogenic risk of soil in the disposal center was 1.02E-06, and the non-carcinogenic hazard index reaches 1.41E+00. Soil carcinogenic risk exceeded the acceptable level of 1E-06 required by the *Technical guidelines for risk assessment of soil contamination of land for construction* (HJ 25.3-2019). The non-carcinogenic hazard index exceeded the acceptable level hazard quotient 1 required by the *Technical guidelines for risk assessment of soil contamination of land for construction* (HJ 25.3-2019). Thus, under the condition of reutilization, the risk control value of Cd in the soil needed to be further discussed.
2. Risk characterization results under the condition of non-utilization
  The health risks of the pollutants in the soil under the condition of non-utilization are shown in Tables 3, 4. It can be seen from Tables 3, 4 that under the condition of non-utilization, the total carcinogenic risk of soil in the disposal center was 1.77E-07, and the non-carcinogenic hazard index reached 6.91E-01. Soil carcinogenic risk does not exceed the acceptable level of 1E-06 required by the *Technical guidelines for risk assessment of soil contamination of land for construction* (HJ 25.3-2019). The non-carcinogenic hazard index did not exceed the acceptable level hazard quotient 1 required by the *Technical guidelines for risk assessment of soil contamination of land for construction* (HJ 25.3-2019). The carcinogenic risk and non-carcinogenic risk of Cd were acceptable under the condition of non-utilization.

**Table 1:** Soil risk results under the condition of reutilization (carcinogenic risk).

**Sampling point**	**Depth**	**Pollutant**	** ${\text{CR}}_{\text{s}}^{\text{ing}}$ **	** ${\text{CR}}_{\text{s}}^{\text{der}}$ **	** ${\text{CR}}_{\text{s}}^{\text{ip}}$ **	** ${\text{CR}}_{\text{s}}^{\text{op}}$ **	** ${\text{CR}}_{\text{s}}^{\text{iv}}$ **	** ${\text{CR}}_{\text{s}}^{\text{sur-ov}}$ **	** ${\text{CR}}_{\text{s}}^{\text{sub-ov}}$ **	** ${\text{CR}}_{\text{s}}^{\text{T-on}}$ **
S3	0.5 m	Cd	-	-	8.47E-07	1.77E-07	-	-	-	1.02E-06

**Table 2:** Soil risk results under the condition of reutilization (non-carcinogenic hazard index).

**Sampling point**	**Depth**	**Pollutant**	** ${\text{HQ}}_{\text{s}}^{\text{ing}}$ **	** ${\text{HQ}}_{\text{s}}^{\text{der}}$ **	** ${\text{HQ}}_{\text{s}}^{\text{ip}}$ **	** ${\text{HQ}}_{\text{s}}^{\text{op}}$ **	** ${\text{HQ}}_{\text{s}}^{\text{iv}}$ **	** ${\text{HQ}}_{\text{s}}^{\text{sur-ov}}$ **	** ${\text{HQ}}_{\text{s}}^{\text{sub-ov}}$ **	** ${\text{HI}}_{\text{s}}^{\text{on}}$ **
S3	0.5 m	Cd	4.36E-01	1.05E-01	7.16E-01	1.49E-01	-	-	-	1.41E+00

**Table 3:** Soil risk results under the condition of non-utilization (carcinogenic risk).

**Sampling point**	**Depth**	**Pollutant**	** ${\text{CR}}_{\text{s}}^{\text{ing}}$ **	** ${\text{CR}}_{\text{s}}^{\text{der}}$ **	** ${\text{CR}}_{\text{s}}^{\text{op}}$ **	** ${\text{CR}}_{\text{s}}^{\text{sur-ov}}$ **	** ${\text{CR}}_{\text{s}}^{\text{sub-ov}}$ **	** ${\text{CR}}_{\text{s}}^{\text{T-on}}$ **
S3	0.5 m	Cd	-	-	1.77E-07	-		1.77E-07

**Table 4:** Soil risk results under the condition of non-utilization (non-carcinogenic hazard index).

**Sampling point**	**Depth**	**Pollutant**	** ${\text{HQ}}_{\text{s}}^{\text{ing}}$ **	** ${\text{HQ}}_{\text{s}}^{\text{der}}$ **	** ${\text{HQ}}_{\text{s}}^{\text{op}}$ **	** ${\text{HQ}}_{\text{s}}^{\text{sur-ov}}$ **	** ${\text{HQ}}_{\text{s}}^{\text{sub-ov}}$ **	** ${\text{HI}}_{\text{s}}^{\text{on}}$ **
S3	0.5 m	Cd	4.36E-01	1.05E-01	1.49E-01	-		6.91E-01

#### Contributive Rates of Exposure Pathways

The risk contribution rates of soil pollutants *via* different exposure pathways under the two conditions of reutilization and non-utilization were calculated, and the results are shown in Supplementary Tables S3–S6. It can be seen from Supplementary Tables S3, S4 that, the carcinogenic and non-carcinogenic risks of Cd mainly came from the oral intake of soil and inhalation of indoor soil particles under the condition of reutilization. It can be seen from Supplementary Tables S5, S6 that the carcinogenic and non-carcinogenic risks of Cd mainly came from oral intake of soil and inhalation of outdoor soil particles under the condition of non-utilization.

### Migration Effects of Heavy Metals and Fluoride in Groundwater

**Figure 4:**
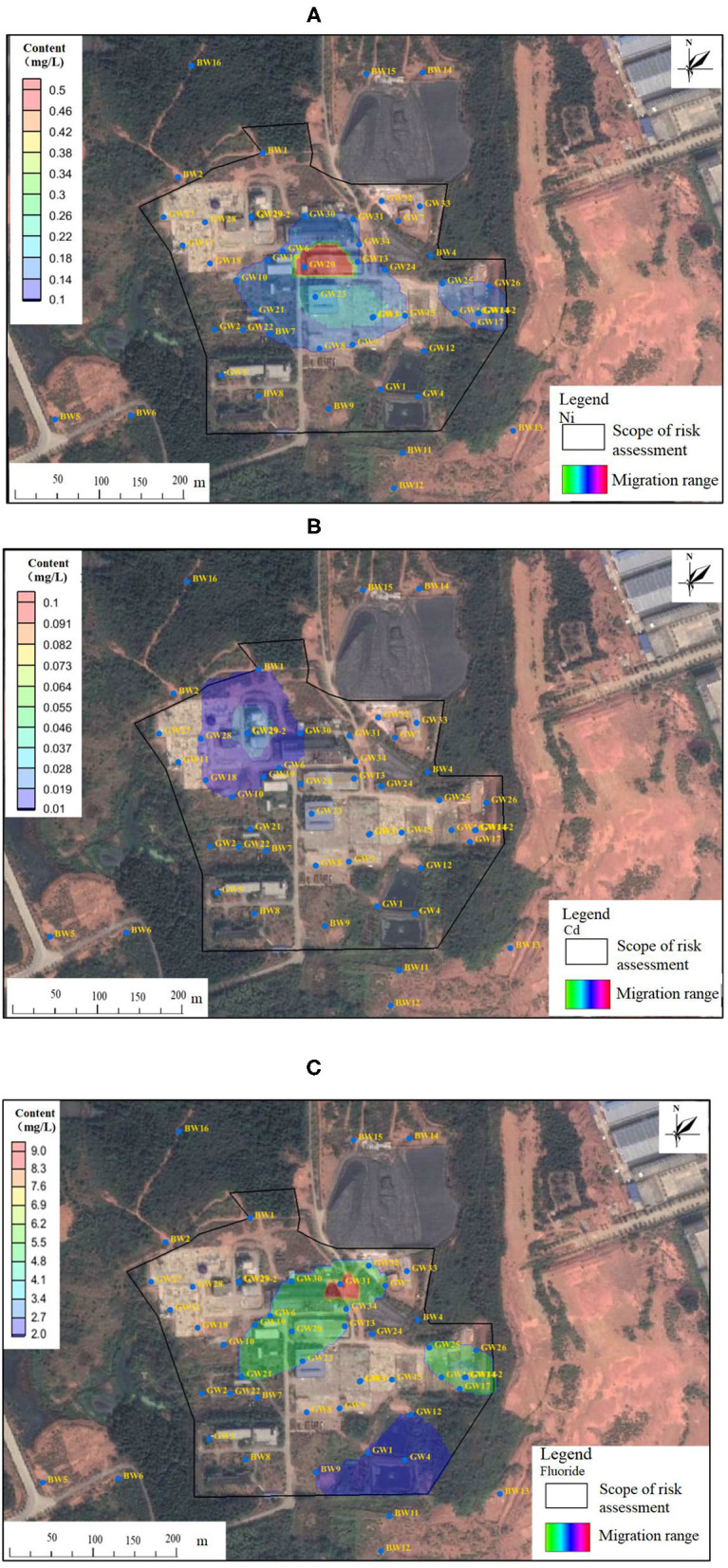
Nickel **(A)**, Cd **(B)**, and fluoride **(C)** plume simulation in the next 50 years.

According to the models and parameters in section Simulation and Prediction Model of Pollutants in Groundwater, the migration of heavy metals and fluoride exceeding groundwater standards (GB/T 14848-2017) was simulated. The migration results of heavy metals (Ni and Cd) and fluoride in the 50-year simulation period are shown in Figure 4. From Figure 4, it can be seen that heavy metals (nickel and Cd) and fluoride plumes were unlikely to migrate and diffuse out of the western and southern site boundaries within 50 years. Fluoride, nickel, and Cd may be greatly affected by the adsorption of soil.

### Risk Management Suggestions

Pollutants that exceeded the corresponding environmental standard values in a single medium in the preliminary and detailed investigation were considered pollutants of concern, which were further subject to health risk assessments under the two land-use plans. For the pollutant whose health risk exceeds the corresponding risk limit, further regulatory measures are necessary, so the recommended control values need to be calculated. The risk control value was not necessary for groundwater, but the risk of groundwater migration and diffusion must be paid attention to. For soil under the condition of non-utilization, there was no need to calculate the recommended risk control values for pollutants. Therefore, the recommended risk control values for pollutants in soil are discussed under the condition of reutilization.

For greater rationality of the calculated risk control value to be directly considered as the risk control value in the disposal center, the calculated risk control value (56 mg/kg for Cd) and the values of the soil control point (0.04 mg/kg for Cd), domestic relevant standards (screening value of 65 mg/kg and intervention value of 172 mg/kg for Cd in the second type of land for construction), and domestic existing cases (65 mg/kg) were compared to put forward the suggested risk control value of soil pollutants under the condition of reutilization-−56 mg/kg for Cd. Under the condition of non-utilization, based on key information of sampling points exceeding standard (GB36600-2018), the engineering quantity of soil risk control in the disposal center was estimated and characterized. The area of soil contaminated by Cd was about 630.58 m^2^, and the amount of pollution was about 1261.16 m^3^. The heavy metal pollution was only distributed in the depth range of 0–2 m, which was between the screening value and the intervention value.

Under the condition of reutilization, the risk of soil Cd was unacceptable and the single area and volume were relatively small, so the control strategy of ectopic excavation and barrier landfill is recommended. For polluted groundwater, the simulation results of pollution migration showed that the speed of pollution migration was very slow, so the control strategy of “monitoring the natural attenuation and long-term monitoring” is suggested. For the landfill, there was no significant leakage in the landfill, but there remained the risk of leakage in the landfill detection investigation. It is suggested that the landfill should be closed and greened as soon as possible, and drainage measures should be taken to avoid the downward migration of pollution caused by rainwater leaching and infiltration.

## Conclusions

In the pollution investigation of groundwater and soils, COD, fluoride, nickel, Cd, lead, and beryllium exceeded the corresponding environmental standard in a single medium. However, whether the contents of pollutants in a single medium exceed the standard or not is not enough for management decisions. It is necessary to further carry out the risk assessment of these pollutants of concern to evaluate the harmful effects. Among them, the carcinogenic and non-carcinogenic risks of Cd in soil exceeded the corresponding risk assessment limits, so Cd needed to be managed specifically, and the recommended control value of Cd is further proposed. Based on concern contaminants identification in preliminary and detailed investigation and further health risk assessment under two land use plans, the area of soil contaminated by Cd was evaluated to be about 630.58 m^2^, and the amount of pollution was about 1261.16 m^3^. The heavy metal pollution was only distributed in the depth range of 0–2 m, which was between the screening value and the intervention value. The suggested risk control value of soil pollutants under the condition of reutilization was 56 mg/kg for Cd. Under the condition of reutilization, the risk of soil Cd was unacceptable and the single area and volume were relatively small, so the control strategy of ectopic excavation and barrier landfill is recommended. Contributive rates analyze of exposure pathways showed that oral intake of soil and inhalation of indoor soil particles were the main pathways contributing to the carcinogenic and non-carcinogenic risks under the two conditions of reutilization and non-utilization. The contaminants of concern identified by the preliminary and detailed investigation, heavy metals and fluoride, are not volatile. Therefore, it was considered that there were no vapor exposure pathways for soil and groundwater to the human body. The risk control value was not necessary for groundwater, but the risk of groundwater migration and diffusion must be paid attention to. The migration of heavy metals and fluoride exceeding groundwater standard (GB36600-2018) were simulated. The migration speed of fluoride, nickel, and Cd in groundwater was slow, and the pollution range of each pollutant changes little in 50 years. For polluted groundwater, the simulation results of pollution migration showed that the speed of pollution migration was very slow, so the control strategy of “monitoring the natural attenuation and long-term monitoring” is suggested. For the landfill leakage risk, the landfill should be closed and greened with drainage measures to avoid the downward migration of pollution caused by rainwater leaching and infiltration.

## Data Availability Statement

The raw data supporting the conclusions of this article will be made available by the authors, without undue reservation.

## Author Contributions

WZ and XW contributed to the conception of the study. WZ and XY performed the experiment. WZ, XY, and JH contributed significantly to analysis and manuscript preparation. WZ and XY performed the data analyses and wrote the manuscript. JH, RL, and ZZ helped perform the analysis with constructive discussions. All authors have read and agreed to the published version of the manuscript.

## Funding

This work was supported by the National Key Research and Development Program of China (2018YFC1800205 and 2018YFC1800200).

## Conflict of Interest

The authors declare that the research was conducted in the absence of any commercial or financial relationships that could be construed as a potential conflict of interest.

## Publisher's Note

All claims expressed in this article are solely those of the authors and do not necessarily represent those of their affiliated organizations, or those of the publisher, the editors and the reviewers. Any product that may be evaluated in this article, or claim that may be made by its manufacturer, is not guaranteed or endorsed by the publisher.
