# Supplementary material for: Investigation and Systematic Risk Assessment in a Typical Contaminated Site of Hazardous Waste Treatment and Disposal
Source: Front Public Health. 2021 Oct 27;9:764788. doi: 10.3389/fpubh.2021.764788 (PMC8578362; doi:10.3389/fpubh.2021.764788)
Supplement: Supplementary file 1 [file Data_Sheet_1.docx]

# Supplementary materials

## S1 The exposure dose of (non-)carcinogenic pollutants via other pathways to be exposed of soil or groundwater

### **Skin contact with soil**

For carcinogenic pollutants, the exposure dose via the skin contact with soil for adults is calculated as Formula (S1).

${DCSER}_{ca}=\frac{\mathrm{SAE}_{a}\times{\mathrm{SSAR}_{a}\times{EF}_{a}\times ED}_{a}\times E_{V}{\times ABS}_{d}}{{BW}_{a}\times{AT}_{ca}}\times{10}^{-6}$ (S1)

where DCSER_ca_ is the exposure dose via the skin contact with soil (carcinogenic effect), kg (soil)·kg^-1^ (body weight)·d^-1^; SAE_a_ is exposed skin surface area for adults, cm^2^; SSAR_a_ is soil adhesion coefficient on adult skin surface, mg·cm^-2^; ABS_d_ is skin contact absorption efficiency factor, dimensionless; E_v_ is frequency of daily skin contact, times d^-1^; The recommended values of SSAR_a_, ABS_d_ and Ev are shown in Table S1. For non-carcinogenic pollutants, Formula (S2) is used to calculate the exposure dose via the skin contact with soil for adults.

${DCSER}_{nc}=\frac{{OSIR}_{a}\times{ED}_{a}\times{EF}_{a}\times{ABS}_{o}}{{BW}_{a}\times{AT}_{nc}}\times{10}^{-6}$ (S2)

where DCSER_nc_ is the exposure dose via the skin contact with soil (non-carcinogenic effect), kg (soil)·kg^-1^ (body weight)·d^-1^; The meanings of EF_a_, ED_a_, and BW_a_ are shown in Formula (1), AT_nc_ in Formula (2), and SAE_a_, SSAR_a_, E_v_ and ABS_d_ in Formula (S1).

### **Inhalation of soil particles**

For carcinogenic pollutants, the exposure dose via the inhalation of soil particles for adults is calculated as Formula (S3).

${PISER}_{ca}=\frac{{PM}_{10}\times{DAIR}_{a}\times{ED}_{a}\times PIAF\times(fspo\times{EFO}_{a}+fspi\times{EFI}_{a})}{{BW}_{a}\times{AT}_{ca}}\times{10}^{-6}$ (S3)

where PISER_ca_ is the exposure dose via the inhalation of soil particles (carcinogenic effect), kg (soil)·kg^-1^ (body weight)·d^-1^; PM10 is inhalable content of particles in air, mg^3^.m^-3^; DAIR_a_ is daily air respiration for adults, m^3^.d^-1^; PIAF - Inhaled soil particles in vivo retention ratio, dimensionless; *fspo* is proportion of outdoor soil particles, dimensionless; *fspi* is proportion of indoor soil particles, dimensionless; EFO_a_ is outdoor exposure frequency for adults, d·a^-1^; EFI_a_ is adult indoor exposure frequency, d·a^-1^. The recommended values of PM_10_, DAIR_a_, PIAF, *fspo*, *fspi*, EFO_a_, EFI_a_ are shown in Table S1. Formula (1) shows the meaning of ED_a_, BW_a_, and AT_ca_. For non-carcinogenic pollutants, Formula (S4) is used to calculate the exposure dose via the inhalation of soil particles for adults.

${PISER}_{nc}=\frac{{PM}_{10}\times{DAIR}_{a}\times{ED}_{a}\times PIAF\times(fspo\times{EFO}_{a}+fspi\times{EFI}_{a})}{{BW}_{a}\times{AT}_{nc}}\times{10}^{-6}$ (S4)

where PISER_nc_ is the exposure dose via inhalation of outdoor soil particles (non-carcinogenic effect), kg (soil)·kg^-1^ (body weight)·d^-1^; The meanings of ED_a_、BW_a_ are shown in Formula (1), AT_nc_ in Formula (2), and PM_10_, DAIR_a_, ED_a_, PIAF, *fspo*, *fspi*, EFO_a_ and EFI_a_ in Formula (S3).

## S2 Toxicity evaluation model (carcinogenic slope factor, reference dose)

The toxic parameter values of pollutants were determined according to Appendix B of *Technical guidelines for risk assessment of soil contamination of land for construction* (HJ 25.3-2019). The respiratory inhalation carcinogenic slope factor (SFi) was calculated by extrapolation based on the respiratory inhalation carcinogenic factor (URF) in Formula (S5). The reference dose of respiratory inhalation (RfDi) was calculated by extrapolation according to the reference concentration of respiratory inhalation (RfC) in Formula (S6).

${SF}_{i}=\frac{\mathrm{IUR}\times{BW}_{a}}{{DAIR}_{a}}$ (S5)

${RfD}_{i}=\frac{\mathrm{RfC}\times{DAIR}_{a}}{{BW}_{a}}$ (S6)

where Sf_i_ is carcinogenic slope factor for respiratory inhalation, (mg (pollutant) kg^-1^ (body weight) d^-1^)^-1^; RfD_i_ is reference dose for respiratory inhalation, mg (pollutant) kg^-1^ (body weight) d^-1^; IUR is carcinogenic factor for respiratory inhalation, m^3^·kg^-1^; RfC is reference concentration for respiratory inhalation, mg·m^-3^. The recommended values of SF_i_, RfD_i_, IUR, and RfC are shown in Table S1. The meaning of BW_a_ is shown in Formula (1), and the meaning of DAIR_a_ is shown in Formula (S3). Skin contact carcinogenic slope factor (SF_d_) is calculated according to oral intake carcinogenic slope factor. Skin contact reference dose (RfD_d_) is calculated according to oral intake reference dose. The skin contact carcinogenic slope coefficient and reference dose were calculated by Formula (S7) and Formula (S8), respectively.

${SF}_{d}=\frac{{SF}_{o}}{{ABS}_{gi}}$ (S7)

${RfD}_{d}={RfD}_{o}\times{ABS}_{gi}$ (S8)

where SF_d_ is carcinogenic slope factor for skin contact, (mg (pollutant) kg^-1^ (body weight) d^-1^)^-1^; SF_o_ is carcinogenic slope factor for oral intake, (mg (pollutant) kg^-1^ (body weight) d^-1^)^-1^; RfD_o_ is reference dose for oral intake, mg (pollutant) kg^-1^ (body weight) d^-1^; RfD_d_ is reference dose for skin contact, mg (pollutant) kg^-1^ (body weight) d^-1^; ABS_gi_ is efficiency factor for digestive tract absorption, dimensionless. The recommended values of SF_d_, SF_o_, RfD_o_, RfD_d_, and ABS_gi_ are shown in Table S1.

## S3 The carcinogenic risks of single pollutant via other exposure pathways for soil or groundwater

The carcinogenic risk of single pollutant in soil via skin contact was calculated as Formula (S9).

${CR}_{dcs}={DCSER}_{ca}\times C_{sur}\times{SF}_{d}$ (S9)

where CR_dcs_ is the carcinogenic risk via skin contact with soil, dimensionless. The meaning of DCSER_ca_ is shown in Formula (S1), SF_d_ in Formula (S7), C_sur_ in Formula (3). The carcinogenic risk of single pollutant in soil via the inhalation of soil particles was calculated as Formula (S10).

${CR}_{pis}={PISER}_{ca}\times C_{sur}\times{SF}_{i}$ (S10)

where CR_pis_ is the carcinogenic risk via the inhalation of soil particles, dimensionless. The meaning of SF_i_ is shown in Formula (S5). The carcinogenic risk of single pollutant of concern via all exposure pathways is calculated as Formula (S11).

${CR}_{i}={CR}_{\mathrm{ois}}+{CR}_{\mathrm{dcs}}+{CR}_{\mathrm{pis}}$ (S11)

where Cr_i_ is total carcinogenic risk of single pollutants in soil ( type i ) via all exposure pathways, dimensionless. The meanings of CR_ois_, CR_dcs_ and CR_pis_ are shown in Formula (3), Formula (S9) and Formula (S10), respectively.

## S4 The hazard quotients of single pollutant via other exposure pathways for soil or groundwater

The hazard quotient of single pollutant in contaminated soil via skin contact was calculated as Formula (S12).

${HQ}_{dcs}=\frac{{DCSER}_{nc}\times C_{sur}}{{RfD}_{d}\times SAF}$ (S12)

where HQ_dcs_ is hazard quotient via skin contact with soil, dimensionless. The meaning of DCSER_nc_ parameter can be found in Formula (S2), RfD_d_ in Formula (S8), C_sur_ in Formula (3), SAF in Formula (4). The hazard quotient of single pollutant in contaminated soil via the inhalation of soil particles was calculated as Formula (31).

${HQ}_{pis}=\frac{{PISER}_{nc}\times C_{sur}}{{RfD}_{i}\times SAF}$ (S13)

where HQ_pis_ is hazard quotient via inhalation of soil particles, dimensionless. The definitions of PISER_nc_ is shown in Formula (S4), RfD_i_ in Formula (6), C_sur_ in Formula (3) and SAF in Formula (4). The hazard index of single pollutant of concern via all exposure pathways is calculated as Formula (40).

${HI}_{i}={HI}_{\mathrm{ois}}+{HI}_{\mathrm{dcs}}+{HI}_{\mathrm{pis}}$ (S14)

where HI_i_ is hazard index of single pollutants in soil ( type i ) via all exposure pathways, dimensionless. The definitions of HQ_ois_, HQ_dcs_ and HQ_pis_ are shown in Formula (4), Formula (S12) and Formula (S13), respectively.

## S5 Recommended values of exposure parameters, main toxicity parameters and Soil property parameters

#### Table S1 Recommended values of the main toxicity parameters of concern pollutants

| CAS | Pollutant | SF_0_ | IUR | RfD_o_ | RfC | ABS_gi_ | ABS_d_ | SF_i_^②^ | SF_d_^②^ | RfD_i_^②^ | RfD_d_^②^ |
| --- | --- | --- | --- | --- | --- | --- | --- | --- | --- | --- | --- |
|  |  | (mg.kg^-1^.d^-1^)^-1^ | (mg.m^-3^)^-1^ | mg.kg^-1^.d^-1^ | mg.m^-3^ | — | — | (mg.kg^-1^.d^-1^)^-1^ | (mg.kg^-1^.d^-1^)^-1^ | mg.kg^-1^.d^-1^ | mg.kg^-1^.d^-1^ |
| 16984-48-8 | Fluoride | - | - | 4.00E-02 | 1.30E-02 | 1.00E+00 | - | - | - | 3.05E-03 | 4.00E-02 |
| 7440-43-9 | Cd | - | 1.80E+00 | 1.00E-03 | 1.00E-05 | 2.50E-02 | 1.00E-03 | 7.67E+00 | - | 2.35E-06 | 2.50E-05 |
| 7440-02-0 | Ni | - | 2.60E-01 | 2.00E-02 | 9.00E-05 | 4.00E-02 | - | 1.11E+00 | - | 2.11E-05 | 8.00E-04 |

Note: ① The parameter values come from Appendix B of *Technical guidelines for risk assessment of soil contamination of land for construction* (HJ 25.3-2019). ② The parameter values are derived from the extrapolation model Formulas B.1 and B.2 of *Technical guidelines for risk assessment of soil contamination of land for construction* (HJ 25.3-2019).

#### Table S2 Soil property parameters

| Layer number | Lithology | Parameters | Unit weight | Mass fraction of organic carbon | Grain density | Total porosity | Moisture content | Pore water volume ratio | Pore air volume ratio |
| --- | --- | --- | --- | --- | --- | --- | --- | --- | --- |
|  |  |  | ρd | foc | ρs | θ | Pws | θws | θas |
|  |  |  | kg/dm^3^ | — | kg/dm^3^ | — | kg_水_/kg_土_ | — | — |
| ① | Plain fill | MIN | 1.51 | 0.0017 | 2.72 | 0.404 | 0.235 | 0.381 | 0.024 |
|  |  | MAX | 1.62 | 0.0045 | 2.72 | 0.445 | 0.253 | 0.386 | 0.063 |
|  |  | MEAN | 1.57 | 0.0031 | 2.72 | 0.423 | 0.244 | 0.383 | 0.040 |
| ② | Aeration zone | MIN | 1.51 | 0.0003 | 2.72 | 0.375 | 0.226 | 0.363 | 0.009 |
|  |  | MAX | 1.7 | 0.0021 | 2.72 | 0.445 | 0.274 | 0.436 | 0.067 |
|  |  | MEAN | 1.585 | 0.0011 | 2.72 | 0.417 | 0.246 | 0.390 | 0.036 |
| ③ | Saturated zone | MIN | 1.52 | 0.0003 | 2.72 | 0.386 | 0.230 | 0.377 | 0.002 |
|  |  | MAX | 1.67 | 0.0014 | 2.72 | 0.441 | 0.289 | 0.439 | 0.047 |
|  |  | MEAN | 1.59 | 0.0006 | 2.72 | 0.415 | 0.253 | 0.403 | 0.024 |

## S6 The risk contribution rates of soil pollutants via different exposure pathways

#### Table S3 The risk contribution rates of soil pollutants via different exposure pathways under the condition of reutilization (carcinogenic risk)

| Sampling point | Depth | Pollutant | PPC_s_^ip^ | PPC_s_^op^ | PPC_s_^Total^ |
| --- | --- | --- | --- | --- | --- |
| S3 | 0.5m | Cd | 82.76 | 17.24 | 100.00 |

#### Table S4 The risk contribution rates of soil pollutants via different exposure pathways under the condition of reutilization (non-carcinogenic hazard index)

| Sampling point | Depth | Pollutant | PPC_s_^ing^ | PPC_s_^der^ | PPC_s_^ip^ | PPC_s_^op^ | PPC_s_^Total^ |
| --- | --- | --- | --- | --- | --- | --- | --- |
| S3 | 0.5m | Cd | 31.01 | 7.50 | 50.89 | 10.60 | 100.00 |

#### Table S5 The risk contribution rates of soil pollutants via different exposure pathways under the condition of non-utilization (carcinogenic risk)

| Sampling point | Depth | Pollutant | PPC_s_^op^ | PPC_s_^Total^ |
| --- | --- | --- | --- | --- |
| S3 | 0.5m | Cd | 100.00 | 100.00 |

#### Table S6 The risk contribution rates of soil pollutants via different exposure pathways under the condition of non-utilization (non-carcinogenic hazard index)

| Sampling point | Depth | Pollutant | PPC_s_^ing^ | PPC_s_^der^ | PPC_s_^op^ | PPC_s_^Total^ |
| --- | --- | --- | --- | --- | --- | --- |
| S3 | 0.5m | Cd | 63.14 | 15.27 | 21.58 | 100.00 |

Note: “PPC_s_^ing^” represents the oral intake pathway, “PPC_s_^der^” represents the skin contact pathway, “PPC_s_^ip^” represents the inhalation pathway of indoor soil particles, “PPC_s_^op^” represents the inhalation pathway of outdoor soil particles, and “PPC_s_^T^” represents the accumulation of contribution rates of various pathways.

## S7 Photoes of the HWDC


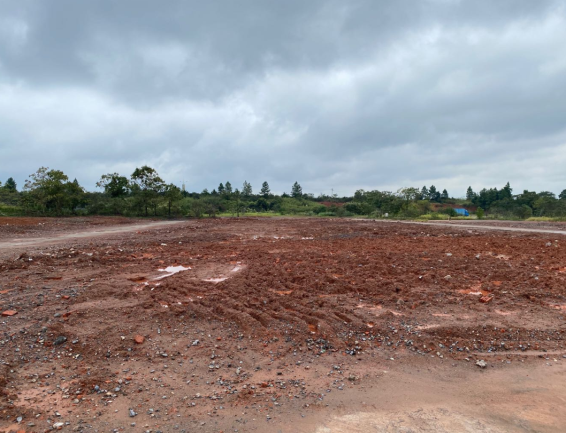

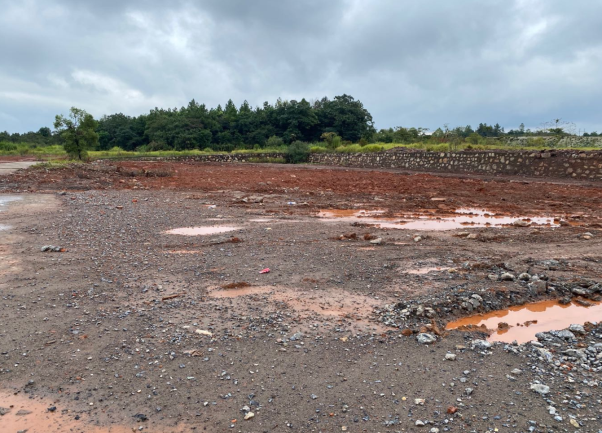


(a) Current incineration workshops (b) Current stabilization/solidification workshops


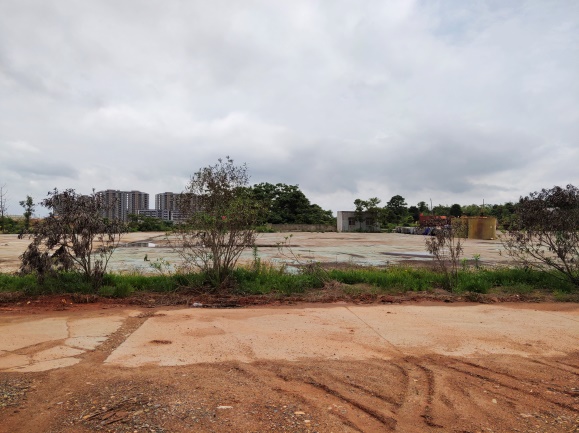

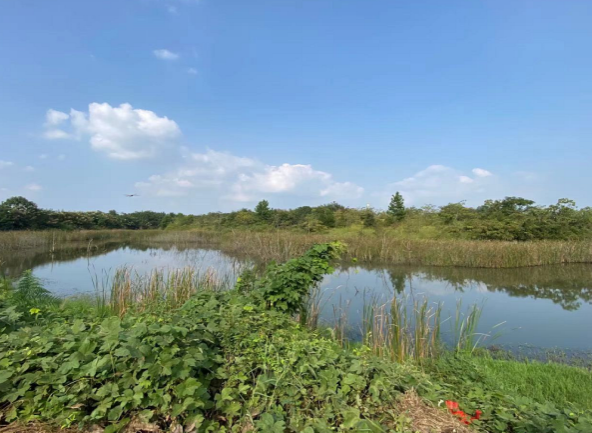


(c) Current material/chemical workshops (d) The pond on the west side in the disposal center


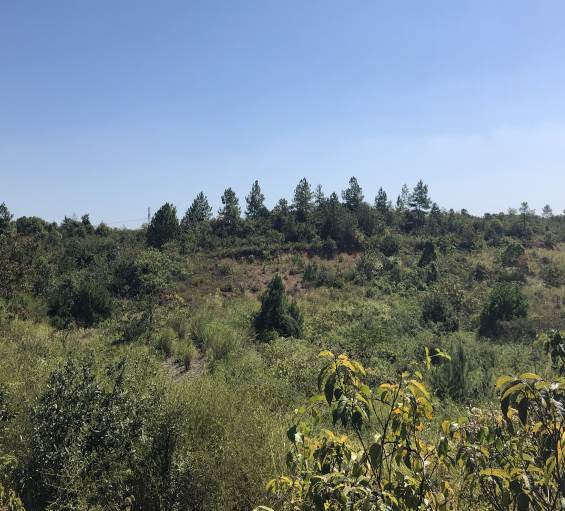

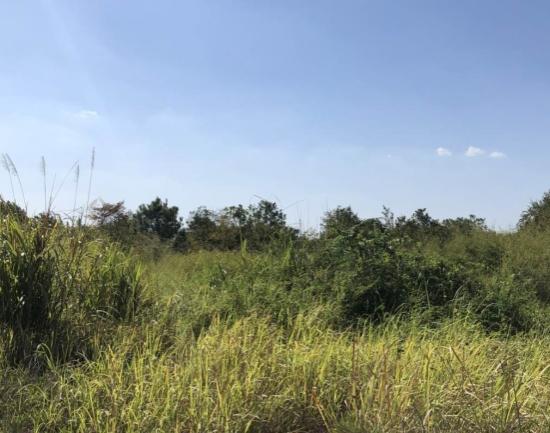


(e) The unutilized landfill area (f) The unused forest land


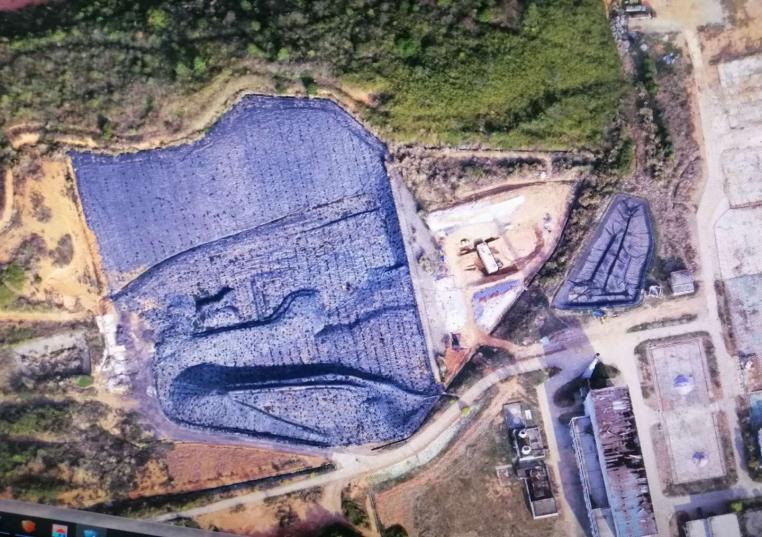

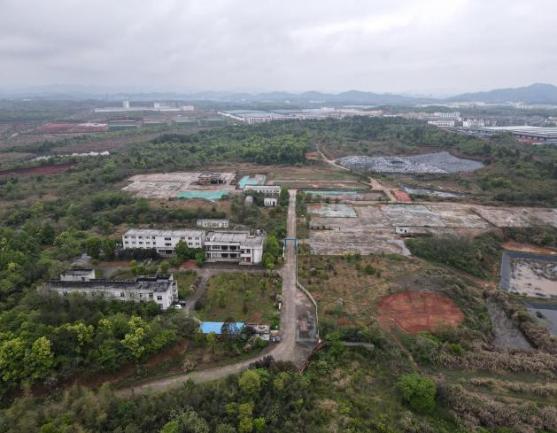


(g) Aerial photograph of the landfill area (h) Aerial photograph of the HWDC

Fig. S1 Photos of the production and management area (a, b, c, and d), the unused land (e and f), the landfill area (g) and the HWDC (h)

## S8 Leakage risk detection of landfill


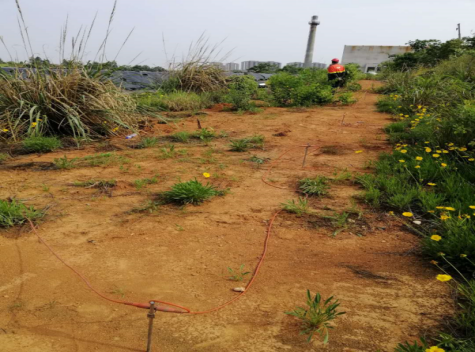


(a) Electrode arrangement for leakage detection in landfill area


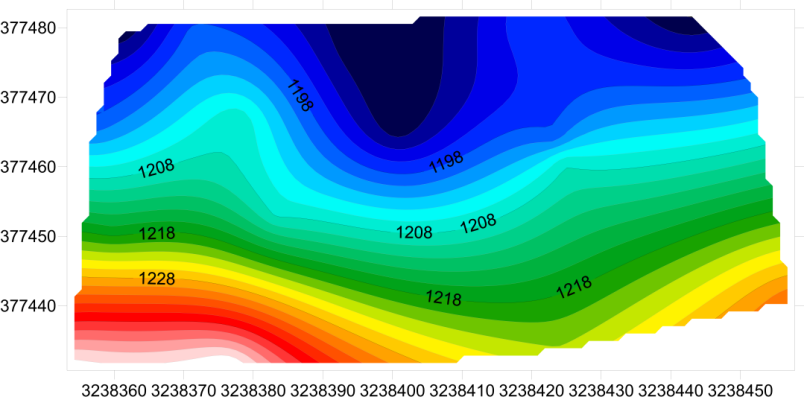


(b) The impedance of the landfill impervious layer

Fig. S2 Leakage risk detection of landfill: electrode arrangement (a) and impedance of layers (b)
